# Characterization of the complete mitochondrial genome of the Dayu yak (*Bos grunniens*)

**DOI:** 10.1080/23802359.2020.1861557

**Published:** 2021-01-19

**Authors:** Shaoke Guo, Haiqing Ma, Qingzhang Zhao, Xita Za, Jian Li, Haiyan Kang, Jie Pei, Xian Guo

**Affiliations:** a Key Laboratory of Yak Breeding Engineering of Gansu Province, Lanzhou Institute of Husbandry and Pharmaceutical Sciences, Chinese Academy of Agricultural Sciences, Lanzhou, People’s Republic of China; b Animal Husbandry and Veterinary Station of Qilian County, Qilian, Qinghai Province of the People’s Republic of China; c Animal Husbandry and Veterinary Station of Xitan Township in Menyuan County, Menyuan, Qinghai Province of the People’s Republic of China

**Keywords:** Illumina sequencing, iterative mapping, mitogenome, neighbor-joining (NJ), phylogenetic analysis

## Abstract

Dayu yak (*Bos grunniens*) is a long-furred yak breed from the Qinghai-Tibetan Plateau, and is highly adapted to local high-altitude and cold environments. In this study, its mitochondrial genome was characterized via high-throughput sequencing technology. The genome is 16,323 bp long with an AT-biased base composition (61.0% A + T; light strand), and harbors the typical set of 37 mitochondrial genes and a noncoding control region. Its gene arrangement is identical to those of other bovid taxa. Phylogenetic analysis suggests that Dayu yak is most closely related to Maiwa, Niangya, Qinghai Plateau, Xueduo and Yushu yaks.

As an iconic symbol of the Qinghai-Tibetan Plateau, domestic yaks (*Bos grunniens*) have long been exploited for meat, milk, transportation and other necessities by local communities (Qiu et al. 2012). Dayu yak is a long-furred yak breed with its distribution mostly restricted to Haibei Tibetan Autonomous Prefecture, Qinghai Province, China, and is highly adapted to local high-altitude and cold environments. To facilitate its genetic assays, its complete mitochondrial genome was assembled and annotated via high-throughput sequencing technology in this study. Furthermore, phylogenetic analysis was also conducted to ascertain its relationship with other taxa within the subfamily Bovinae. The annotated genomic sequence has been deposited into GenBank under the accession number MT649465.

The blood sample of Dayu yak were collected from Yeniugou Township, Qilian County, Haibei Tibetan Autonomous Prefecture, Qinghai Province, China (38.35°N, 99.34°E). The voucher specimen (DAYU20200528) is stored in the Key Laboratory of Yak Breeding Engineering of Gansu Province, Lanzhou Institute of Husbandry and Pharmaceutical Sciences of CAAS (Lanzhou, Gansu Province, China). Total genomic DNAs were isolated and purified using the QIAamp DNA Blood Mini Kit (Qiagen, CA, USA). Following the library preparation, high-throughput sequencing was carried out with the Illumina HiSeq X^TM^ Ten Sequencing System (Illumina, CA, USA) by Annoroad Gene Technology (Beijing, China). Totally, 16.03 M raw reads of 150 bp were obtained, and were used to assemble the mitochondrial genome with MITObim v1.9 (Hahn et al. 2013); the reference sequence (JQ692071) was previously published by Qiu et al. (2012). Annotation of the mitochondrial genome was done in Geneious R11 (Biomatters Ltd., Auckland, New Zealand) by comparing with those of other bovid taxa.

The mitochondrial genome of Dayu yak is 16,323 bp long with an A + T-biased base composition (33.7% A, 25.8% C, 13.2% G and 27.3% T; ‘light strand’), and harbors the typical set of 37 animal mitochondrial genes (13 protein-coding genes, 22 tRNAs and two rRNAs) and one non-coding control region. Its gene arrangement is identical to those of other bovid taxa. The PCGs start with the typical ATA (*ND2, ND3* and *ND5*) or ATG (the 10 other PCGs) initiation codons, and end with TAG (*ND2*), the incomplete T (*COX3, ND3* and *ND4*) or TAA (the nine others) termination codons. The tRNAs range in size from 60 (*tRNA-Ser^AGN^*) to 75 bp (*tRNA-Leu^UUR^*) with a total length of 1509 bp. The two rRNAs are 957 bp (*12S rRNA*) and 1,571 bp (*16S rRNA*) long, respectively, and are separated by *tRNA-Val*. The control region is 893 bp long, and is present between *tRNA-Pro* and *tRNA-Phe*. Besides, a 31-bp-long origin of the L-strand replication is identified between *tRNA-Asn* and *tRNA-Cys*.

To investigate the relationship of Dayu yak with 42 other taxa within the subfamily Bovinae, a neighbor-joining phylogenetic tree was reconstructed using the concatenated sequences of all 13 protein-coding genes (alignment size: 11,370 bp) with MEGA7 (http://www.megasoftware.net/) (Hall 2013; Kumar et al. 2016) (Figure 1). Bootstrap support values were estimated from 1000 random samplings. The outgroup taxa included in the phylogenetic analysis are three species from the subfamily Caprinae, i.e. *Hemitragus jayakari* (FJ207523) (Hassanin et al. 2009), *Naemorhedus goral* (JX188255) (Yang et al. 2013) and *Ovis ammon* (KX609626) (Mao et al. 2017). The result suggests that Dayu yak is more closely related to five local yak breeds (Maiwa, Niangya, Qinghai Plateau, Xueduo and Yushu yaks) than to the other 37 taxa within the subfamily Bovinae (Figure 1).

**Figure 1.**
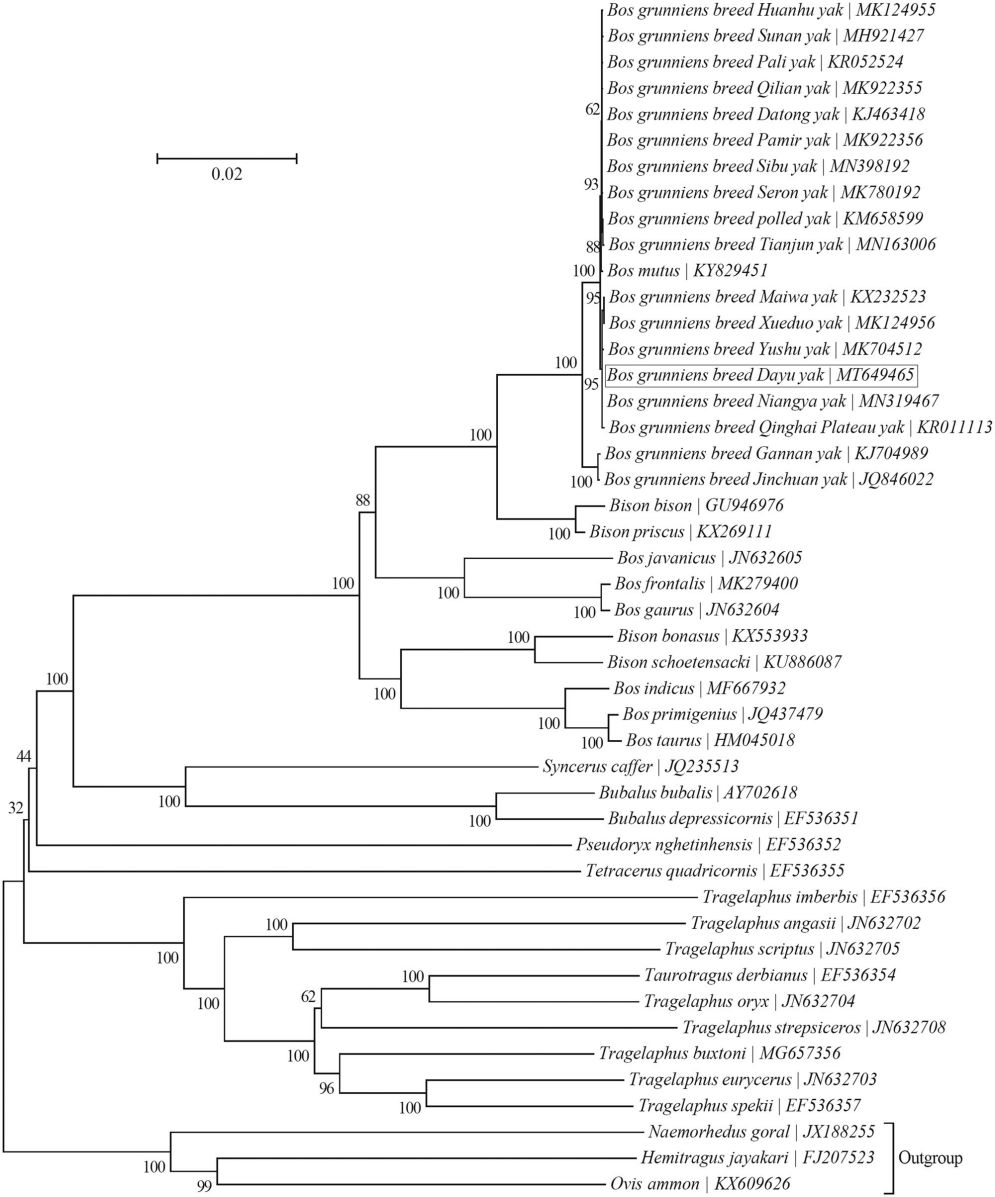
Phylogeny of the subfamily Bovinae based on the neighbor-joining analysis of the concatenated sequences of 13 mitochondrial protein-coding genes (alignment size: 11,370 bp). The bootstrap values next to the nodes are based on 1000 random samplings. Three species within the subfamily Caprinae were included as outgroup taxa.

## Data Availability

The data that support the findings of this study are openly available in GenBank of NCBI at https://www.ncbi.nlm.nih.gov, reference number MT649465.
